# Postintubation tracheal stenosis 35 years after neonatal resuscitation

**DOI:** 10.1016/j.ijscr.2020.02.030

**Published:** 2020-02-19

**Authors:** Yagasaki Hidehiko, Mitsutomo Kohno, Madoka Nito, Naohiro Aruga, Kana Oiwa, Tomoki Nakagawa, Ryota Masuda, Masayuki Iwazaki

**Affiliations:** Division of Thoracic Surgery, Department of Surgery, Tokai University School of Medicine, Isehara, Japan

**Keywords:** Postintubation tracheal stenosis, Endotracheal treatment, Neonatal resuscitation

## Abstract

**Introduction:**

Postintubation tracheal stenosis involves granulation or cicatrization of the tracheal epithelium. It is progressive and can become life-threatening within a few months after extubation.

**Presentation of case:**

We here report a case of tracheal stenosis with a delayed manifestation, presenting 35 years after endotracheal intubation for neonatal resuscitation. A female patient complained of dyspnea during pregnancy. Bronchoscopy revealed 75% constriction of the tracheal lumen by cicatrization, from the 2nd to 4th tracheal rings. After child-birth, the scar tissue was ablated using argon plasma coagulation.

**Discussion:**

The patient had no significant medical history, such as severe airway infection or cervical/chest trauma, which might have caused the circumferential cicatricial tracheal stenosis, other than the endotracheal intubation she had undergone for neonatal resuscitation. Therefore, we considered this to reflect postintubation tracheal stenosis with delayed manifestation.

**Conclusion:**

Delayed postintubation tracheal stenosis should be taken into consideration, when a patient suffers from suffocating tracheal stenosis.

## Highlights

• Granulation or cicatrization on the tracheal lumen increases after injury or ischemia caused by tubes or cuffs, results in narrowing of the trachea within a few months after extubation.
• We have described a case of tracheal stenosis that manifested 35 years after endotracheal intubation for neonatal resuscitation.
• The scar tissue was ablated using argon plasma coagulation, and no recurrence has been observed for more than 3 years.
• Delayed postintubation tracheal stenosis should be taken into consideration, when a patient suffers from suffocating tracheal stenosis.

## 1 Introduction

Posintubation tracheal stenosis is a suffocating and life-threatening condition, first reported by Macewen in 1880 [1]. Granulation or cicatrization on the tracheal lumen increases after injury or ischemia caused by tubes or cuffs, results in narrowing of the trachea late after extubation [2]. Inadequate tube size, excessive high cuff pressure, long intubation periods, intubation of newborn babies and infants, in combination with infection, are considered to be risk factors [3]. Improvement in the shape and materials of tubes and cuffs, and in the management of endotracheal tubes, has reduced the incidence of postintubation tracheal stenosis. However, it is still the most frequent cause of airway constriction after malignant/benign tracheal tumors [4]. The symptoms of postintubation tracheal stenosis usually appear within a few months after extubation [2]; however, here we report a case of this condition which manifested extremely late after extubation and necessitated treatment. This work has been reported in line with the SCARE criteria [5].

## 2 Case report

**Fig. 1:**
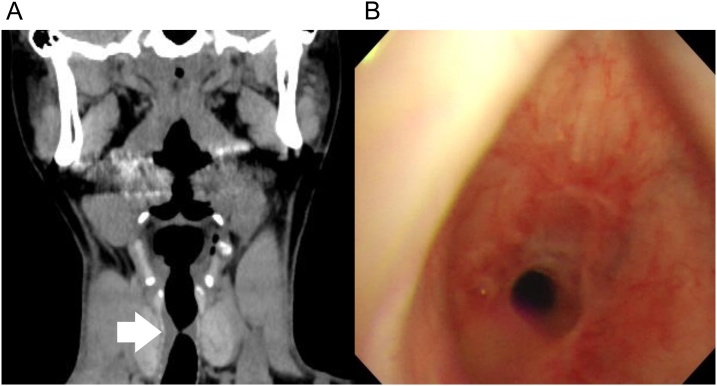
(A) Cervical/chest computed tomography scan (coronal view) taken at the onset, showing tracheal stenosis at the level of the 2nd to 4th rings (arrow). (B) Bronchoscopy image, showing a 75% constriction of the tracheal lumen due to cicatrization.

A 35-year-old female complained of dyspnea during pregnancy with her second child. She had no obesity or complications during pregnancy. Stridor were heard on auscultation. Computed tomography (CT) scan was delayed after delivery despite symptom onset in the third trimester of pregnancy; it was believed that CT imaging would have little influence on the fetus. Cervical and chest CT scan showed upper tracheal stenosis, of a length of 18 mm (Fig. 1A). Video bronchoscopy revealed circumstantial cicatricial stenosis of the trachea at the level of the 2nd to 4th rings, with narrowing resulting in 75% constriction (i.e., leaving 25% of the tracheal lumen unobstructed). The tip of the bronchoscope could narrowly pass through the stenotic tracheal lesion, but resulted in her choking. The patient’s mother recalled that she was a low birth-weight infant (around 1000 g) and underwent neonatal resuscitation via endotracheal intubation and mechanical ventilation for a few weeks. No tracheostomy had been made. However, further detail, such as the size of the endotracheal tube was unknown.

**Fig. 2:**
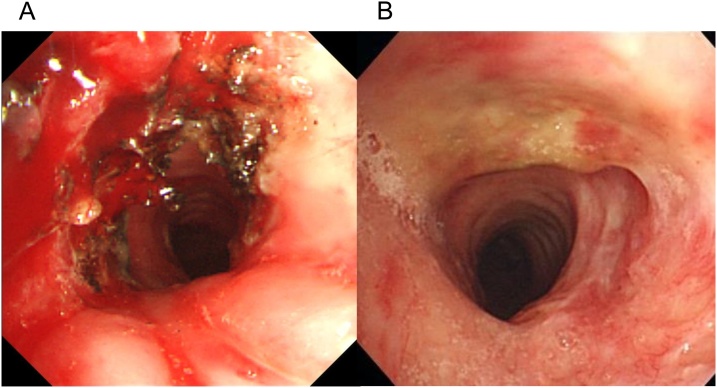
(A) Bronchoscopy image showing cicatrization tissue in the tracheal lumen ablated by bronchoscopic argon plasma coagulation. (B) Bronchoscopy showing successful dilation of the tracheal lumen at 2 months after endotracheal treatment.

The patient did not opt for surgical treatment because she had to take care of her baby; therefore, we chose endotracheal treatment. After topical anesthesia was achieved by spraying 3–4 ml of 2% lidocaine onto the pharyngolarynx, she was sedated with intravenous injection of midazolam (0.06 mg/kg) and placed in a supine position. A 3-mm flexible fiberoptic bronchoscope (Olympus Optical, Tokyo, Japan) was introduced orally; the tracheal stenotic lesion was visualized, and a 6-mm O.D. uncuffed endotracheal tube was guided over the shaft of the pre-inserted fiberscope. The tube could narrowly pass over the stenotic lesion, with some resistance. Thereafter the endotracheal tube was repeatedly changed to one-size larger tube, and finally, an 11.9-mm O.D. (I.D. 8.0 mm) tracheal tube was passed over the stenosis. A video bronchoscope (O.D. 6 mm; Olympus Optical, Tokyo, Japan) was used and a flexible probe of an endoscopic argon plasma coagulation (APC) system (ERBE Elektromedizin, Tübingen, Germany) was introduced via the forceps channel. The scar tissue on the 2nd to 4th ring was ablated until the tracheal lumen was satisfactorily dilated (Fig. 2). Treatment was uneventfully completed in approximately 2 h.

**Fig. 3:**
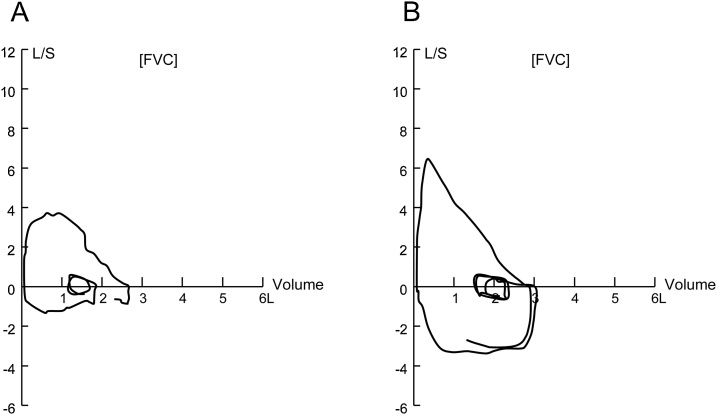
Flow volume curves (A) before and (B) 4 months after endotracheal dilation therapy, showing marked improvement of peak expiratory flow.

Bronchoscopy showed that the tracheal lumen was adequately dilated, with slight erosion of the luminal wall at 2 months after the treatment. Pulmonary function tests and flow volume curves before and after treatment showed marked improvement of the expiratory peak flow. The patient was free of complaints at follow-up at three years after the treatment (Fig. 3).

## 3 Comment

Postintubation tracheal stenosis usually occurs within a few months after extubation. Papla et al. has reported 46 cases of postintubation tracheal stenosis, for which the time interval between primary intubation and diagnosis ranged from 3 weeks to 2 years (mean: 24 weeks) [2]. However, in the present case, the stenosis manifested 35 years after the intubation episode. Nonetheless, the stenosis might have existed even before clinical manifestation. As a student, she experienced dyspnea on running and often missed her physical education class. Owing to the elevation of diaphragm during pregnancy, and because of the reduced pulmonary function, symptoms of respiratory distress may have exacerbated. She had no medical history, such as external throat injury or surgery for a cervical tumor. Histopathological examination of the tracheal stenosis specimen revealed no specific inflammation, such as polychondritis, sarcoidosis, papillomatosis, or Wegener’s granulomatosis. Moreover, she had no history of severe infection of the pharynx, larynx, or lower respiratory tract; histopathological findings indicated no fungal infection or tuberculosis. Therefore, we considered this case as tracheal stenosis caused by intubation during neonatal resuscitation.

The patient was asymptomatic for gastroesophageal reflux disease, such as heartburn. However, she never underwent an upper gastrointestinal endoscopy, and the preoperative workup did not include ambulatory esophageal three-site pH monitoring, which revealed pathologic levels of gastroesophageal reflux during horizontal decubitus and supraesophagic reflux. Therefore, the possibility that the patient’s tracheal stenosis was caused by gastroesophageal reflux disease cannot be denied.

An uncuffed endotracheal tube is generally used for neonatal resuscitation; thus, the tube itself, rather than a cuff, might have injured the tracheal epithelium and/or cartilage in this case [6]. Endotracheal injury may be combined with infection. Cicatrization may have existed for a long time, or may have progressed slowly over the long period, and symptoms may only have arisen when she became pregnant and developed a restrictive respiratory defect. Because no significant symptoms presented during her first pregnancy, two years ago, the tracheal stenosis may have progressed in this duration.

For treatment, we chose endotracheal treatment as this was the patient’s wish at this time [7,8]; however, more radical surgical treatment, such as segmental resection/anastomosis of the trachea or stenting will be necessary should there be a recurrence [6]. Dilation with tracheal tubes and an endoscopic argon plasma coagulation (APC) was initiated. First, after ensuring airway maintenance with an endotracheal tube, risk of suffocation due to bleeding, etc. was mitigated. We believe that a combination of ablation and dilatation is often useful for the treatment of airway obstruction. Because mitomycin-C treatment might be less effective for extremely hard scar constriction, we did not use mitomycin-C for adjunctive therapy this time. The patient had no complaints, and the peak flow value by pulmonary function at three-year follow-up was the same as that after treatment without any adjunctive therapy.

In conclusion, we have described a case of tracheal stenosis that presented 35 years after endotracheal intubation for neonatal resuscitation. When a patient presents with airway constriction, postintubation tracheal stenosis with a delayed manifestation should be considered as a possible cause.

## Sources of funding

This research received no specific grant from any funding agency in the public, commercial, or not-for-profit sectors.

## Ethical approval

Case reports are exempted from ethical approval according to our institution policies.

## Consent

Written informed consent was obtained from the parent for publication of this case report and accompanying images.

## Author contribution

All authors in this manuscript contributed to the interpretation of data, drafting and writing of this manuscript. Hidehiko Yagasaki is first author of this paper. Mitsutomo Kohno is corresponding author of this paper. Hidehiko Yagasaki, Mitsutomo Kohno, Tomoki Nakagawa, and Ryota Masuda conceived and designed the study and drafted the manuscript. Hidehiko Yagasaki, Mitsutomo Kohno, Madoka Nito, Naohiro Aruga, and Kana Oiwa were engaged in patient’s care in our hospital including surgery. Masayuki Iwazaki contributed to study concept, and review of the final manuscript and submission of the paper. All the authors read and approved the final manuscript.

## Registration of research studies

This is no research study.

## Guarantor

Mitsutomo Kohno.

## Provenance and peer review

Not commissioned, externally peer-reviewed.

## Declaration of Competing Interest

None of the authors have any commercial or financial involvement in connection with this study that represents or appears to represent any conflicts of interest.
